# GMS-YOLO11n: A Sheep Detection Model for Challenging Fixed-View Farm Conditions Integrating Spatially Gated Structural Enhancement and Multi-Scale Attention

**DOI:** 10.3390/ani16152351

**Published:** 2026-08-01

**Authors:** Wenbo Yu, Ruoya Xie, Yongqi Liu, Zhi Xue, Zhengpeng Yang, Wenqiang Han

**Affiliations:** 1 College of Mechanical and Electrical Engineering, Inner Mongolia Agricultural University, Hohhot 010018, China; 2 Inner Mongolia Engineering Research Center for Intelligent Equipment for the Entire Process of Forage and Feed Production, Hohhot 010018, China

**Keywords:** sheep detection, object detection, YOLO11n, spatial gating, multi-scale attention, fixed-view farm monitoring

## Abstract

Accurate sheep detection supports counting, tracking, behavior analysis, and health monitoring in intelligent livestock farming. However, occlusion, scale variation, nighttime low light, and background interference can cause missed detections and inaccurate localization. This study proposes GMS-YOLO11n, an improved YOLO11n-based detector for fixed-view sheep monitoring. A spatially gated bottleneck convolution module was introduced at key backbone downsampling stages to strengthen local structural cues, while a multi-scale attention fusion module combined deep semantic information with shallow-to-intermediate details through feature alignment, cross-scale attention, and channel-wise learnable gating. A dataset of 3531 images of Small-tailed Han sheep, containing 8167 annotated instances, was used for evaluation. Across six independent runs, GMS-YOLO11n achieved precision, recall, F1-score, mAP@0.5, and mAP@0.5:0.95 of 93.62% ± 0.12%, 91.03% ± 1.37%, 92.30% ± 0.75%, 96.53% ± 0.49%, and 76.32% ± 0.57%, respectively. Compared with the COCO-pretrained YOLO11n baseline, the corresponding improvements were 1.05, 4.38, 2.79, 2.76, and 6.92 percentage points. Subset analysis showed mAP@0.5:0.95 gains of 9.83 and 7.62 percentage points under nighttime low-light and obvious-occlusion conditions. These results demonstrate improved detection performance under the challenging visual conditions represented in the evaluated single-farm, fixed-view dataset; broader generalization requires external validation.

## Simple Summary

Sheep detection is an important part of intelligent farm management because it supports automatic counting, individual tracking, behavior observation, and health monitoring. However, accurately detecting sheep can be difficult when animals gather closely together, partially block one another, appear at different distances from the camera, or are recorded under poor lighting and complex backgrounds. To address these challenges, we developed GMS-YOLO11n, a new computer vision model for automatic sheep detection. Our approach improves the original YOLO11n model by strengthening important visual features, such as sheep edges, body contours, and local textures, while combining information from different image scales. In the current within-farm evaluation, the proposed model reduced missed detections and improved bounding-box localization, particularly in the nighttime low-light and obvious-occlusion subsets. These findings are limited to the fixed-view surveillance conditions represented in the present dataset and should not be interpreted as evidence of general performance across different farms, breeds, camera devices, or viewpoints. External validation is required before the model can be considered suitable for broader farm deployment.

## 1. Introduction

With the development of artificial intelligence, the Internet of Things, and intelligent sensing technologies, precision livestock farming has become an important part of the digital transformation of modern agriculture [1]. In sheep farming, the continuous and non-contact acquisition of visual information supports individual identification, flock counting, behavior analysis, health monitoring, and welfare assessment. Compared with conventional contact-based methods, such as ear tags and radio-frequency identification (RFID), computer vision-based sheep detection does not require animal-mounted devices and can provide richer spatial and behavioral information [2,3,4]. Therefore, an accurate and computationally efficient sheep detector is important for automated management under farm surveillance conditions.

Sheep detection under fixed-view farm surveillance remains challenging. Sheep are often observed in groups, and mutual occlusion, body overlap, and boundary contact between adjacent individuals may lead to missed detections and inaccurate bounding-box localization [5]. Within a fixed camera view, the distance between sheep and the camera can vary considerably. Consequently, near-field large targets and distant small targets may appear simultaneously in the same image, increasing the demands on the model’s multi-scale feature representation capability [6]. In addition, day–night illumination variations, fence occlusion, ground textures, and backgrounds with colors similar to sheep wool can reduce the visual contrast between sheep and their surroundings, thereby further complicating detection [7]. In intelligent sheep farming applications, missed detections and localization errors may also reduce the reliability of downstream tasks, including sheep counting, individual tracking, behavior recognition, and health monitoring [8]. Therefore, improving model recall and bounding-box localization accuracy under the occlusion, scale variation, illumination changes, and background interference represented in fixed-view monitoring scenarios remains an important issue in sheep detection.

In recent years, deep learning-based object detection methods have been widely applied to livestock detection and behavior recognition. The YOLO series is commonly used in livestock and poultry detection, sheep behavior recognition, and dynamic counting because of its end-to-end framework and high inference efficiency. Guo et al. proposed FESS-YOLOv8n, which combined multi-scale attention with a coordinated spatial–channel strategy for sheep behavior detection. However, its detection accuracy decreased in high-density flocks with severe occlusion [9]. Cheng et al. developed a YOLOv5-based sheep behavior recognition network and examined the influence of training-data characteristics on model performance. Their results showed that differences between the training and test data distributions could substantially affect model generalization [10]. Cao et al. combined an improved YOLOv5x-ECA detector with DeepSort for dynamic sheep counting. Although the method improved detection and tracking performance, occlusion and target overlap still caused missed detections, false detections, and counting errors [11]. These studies demonstrate the potential of deep learning for sheep monitoring, but further improvement is required under fixed-view conditions involving occlusion, scale variation, illumination changes, and background interference.

To enhance target representation in complex scenes, researchers have explored attention mechanisms, multi-scale feature fusion, and lightweight structural designs. Attention mechanisms can assign adaptive weights along the channel or spatial dimension, thereby emphasizing discriminative target responses and suppressing irrelevant background interference [12,13]. The Feature Pyramid Network (FPN) improves multi-scale object detection by combining semantic and detailed information from different feature levels [14], whereas the Path Aggregation Network (PAN) further strengthens the transmission of low-level localization information to high-level semantic features [15]. These multi-scale fusion structures and their variants have been widely used in detection tasks involving small objects and marked scale changes [16]. In livestock farming scenarios, where models are often expected to perform continuous inference on edge devices or surveillance equipment, lightweight backbones, depthwise separable convolutions, and compact detection architectures have also attracted increasing attention, with the aim of balancing detection accuracy, model size, and inference speed [17]. For instance, Pan et al. proposed Chicken-YOLO for detecting caged chickens under low-light and severe-occlusion conditions. By incorporating multi-scale edge information extraction and contextual fusion, the model enhanced local contour perception and improved detection robustness in complex environments [18].

Despite these advances, research specifically addressing sheep detection under the combined effects of occlusion and scale variation remains limited [19]. Existing YOLO-based detectors commonly rely on conventional FPN or PAN pathways for multi-scale feature fusion. Although these structures enable progressive information transfer among feature levels, they may not fully capture the interaction between high-level semantic information and shallow structural details. When sheep are occluded, partially visible, or have weak boundaries, high-level features may retain category-level information but lack detailed edge and contour cues. In contrast, shallow features contain richer spatial information but are more sensitive to background textures and illumination changes [20]. Moreover, simply adding attention modules or lightweight convolutional structures may not provide consistent improvements unless the resulting features are effectively integrated across scales. These limitations motivate a detection framework that strengthens local structural representation and promotes regulated cross-scale interaction while maintaining moderate computational complexity [21].

To address these issues, this study proposes GMS-YOLO11n, an improved sheep detector based on the lightweight YOLO11n architecture. The proposed model retains the original detection framework and introduces two targeted modifications. First, a spatially gated bottleneck convolution module, termed SGBC, is inserted at selected backbone downsampling stages. SGBC combines lightweight bottleneck convolution with spatial gating to strengthen local structural information, including sheep edges, contours, local textures, and contact regions between adjacent animals. Second, a multi-scale attention fusion module, termed MSAMFusion, is introduced at the entrance of the neck. The module aligns features from different scales and integrates high-level semantic information with shallow-to-intermediate details through cross-scale attention and channel-wise learnable gating. The learnable gate regulates the contribution of the enhanced representation during optimization. The model was evaluated using recording-session-level partitions derived from six independent 24 h recording sessions conducted on different dates at a single sheep farm. Complete recording sessions were assigned exclusively to the training, validation, or test set to prevent temporally adjacent frames from the same continuous recording period from being distributed across different subsets. Therefore, the present study evaluates detection performance within the monitored fixed-view farm domain rather than generalization across different farms, sheep breeds, imaging devices, or camera viewpoints.

## 2. Materials and Methods

### 2.1. Dataset Construction and Analysis

Publicly available vision datasets for livestock remain limited, and standardized datasets specifically developed for sheep detection are still scarce [22]. Therefore, video data were collected at the breeding base of Inner Mongolia Beiqi Yaoxie Co., Ltd. (Hohhot, China). The study involved 63 Small-tailed Han sheep aged 1–2 years that were housed indoors under routine farm conditions. Videos were recorded using a DS-2CD3T56FWDV2-I5 network camera (Hangzhou Hikvision Digital Technology Co., Ltd., Hangzhou, China) installed outside the sheep pen. The camera was positioned approximately 10–20 cm above the average shoulder height of the sheep and 1–2 m from the main monitored area. Six independent 24 h recording sessions were conducted on six different dates, resulting in a total raw recording duration of 144 h. Each session covered both daytime and nighttime periods and included variation in illumination, animal position, flock distribution, target scale, mutual occlusion, fence interference, partial visibility, and background appearance.

**Figure 1:**
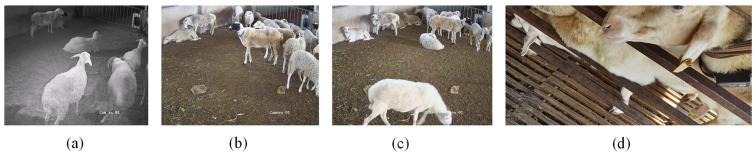
Representative scenes from the sheep detection dataset. The examples show typical detection scenarios in real farm environments, including: (**a**) nighttime low-light conditions; (**b**) flock aggregation and scale variation; (**c**) daytime natural-light conditions; and (**d**) fence occlusion, partially visible sheep, and complex background interference.

After video collection, irrelevant footage and invalid segments were removed using Adobe Premiere Pro 2019. The retained recordings were divided into shorter clips to facilitate frame extraction and data processing. Frames were sampled at intervals of 50 s using OpenCV in Python (v3.12.3) and saved in JPG format. After further screening, 3531 valid images with an original resolution of 2560 × 1920 pixels were retained. The dataset included mutual and partial occlusion, target-scale variation, nighttime low-light conditions, fence occlusion, partially visible sheep, and background interference. Representative examples are shown in Figure 1.

All images were manually annotated with bounding boxes using LabelImg (v1.8.6). One trained annotator completed the initial annotations, which were subsequently reviewed and corrected by two additional researchers. Sheep was the only detection class, and the annotation files were saved in standard YOLO format. A visible-box annotation protocol was adopted, in which each bounding box enclosed only the visible extent of the sheep and did not extrapolate its occluded full-body extent. No fixed percentage-based visibility threshold was applied. Operationally, an occluded sheep was annotated only when (1) the visible region could be unambiguously assigned to a single individual and (2) the visible extent provided sufficiently continuous and distinguishable boundaries in both the horizontal and vertical directions to allow all four sides of the visible-region bounding box to be placed consistently. Instances showing only isolated wool patches, very small disconnected body fragments, or visible regions that could not be reliably separated from adjacent sheep were excluded. Ambiguous cases were reviewed by the two additional researchers before the annotations were finalized. The complete dataset contained 8167 annotated sheep instances.

To reduce temporal redundancy and prevent adjacent or near-duplicate frames from the same continuous recording period from being assigned to different subsets, the dataset was partitioned at the recording-session level. Four complete sessions were assigned to the training set, one to the validation set, and one to the test set. All video clips and sampled frames from the same session remained in the same subset. The training, validation, and test sets contained 2824, 353, and 354 images and 6463, 848, and 856 annotated instances, respectively. Thus, the approximate 8:1:1 ratio refers to the final image counts, whereas the recording sessions were allocated at a ratio of 4:1:1, as summarized in Table 1. Because all sessions were collected from the same farm and sheep breed but from different monitored pens under the same primary fixed-camera configuration, the evaluation represents within-domain performance across independent recording periods and pen environments rather than cross-farm, cross-breed, cross-device, or cross-view generalization.

**Figure 2:**
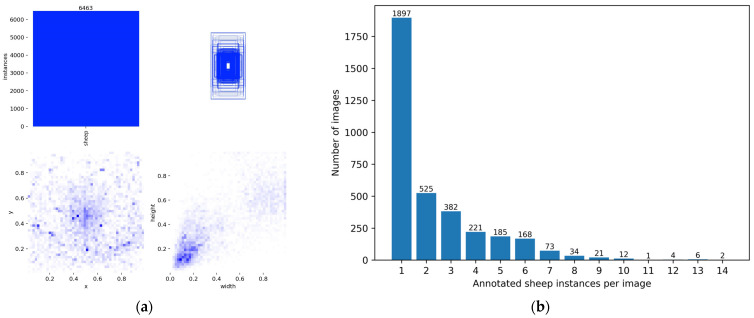
Annotation statistics of the sheep detection dataset: (**a**) class count and normalized distributions of bounding-box centers and sizes in the training subset (2824 images and 6463 instances); and (**b**) distribution of annotated sheep instances per image in the complete dataset (3531 images and 8167 instances).

After dataset partitioning, image-level attributes were assigned according to illumination condition and occlusion degree. A total of 2342 images were classified as daytime natural-light scenes, accounting for 66.33% of the dataset, whereas 1189 images were classified as nighttime low-light scenes, accounting for 33.67%. Regarding occlusion, 805 images were classified as having no obvious occlusion, and 2726 images were classified as having obvious occlusion, accounting for 22.80% and 77.20%, respectively. Obvious occlusion included mutual occlusion between sheep, obstruction by fences or people, foreground sheep obscuring background sheep, and partial target visibility. Illumination and occlusion were treated as independent image-level attributes; therefore, each image received one illumination label and one occlusion label. These labels were used to describe the visual composition of the current dataset and to support the subsequent stratified evaluation. The complete dataset contained an average of 2.31 annotated instances per image, with a median of 1 and a range of 1–14. Most images contained one to three sheep. As shown in Figure 2, the normalized distributions of target centers and bounding-box sizes in the training subset covered a broad range of positions and scales, including large nearby sheep, small distant sheep, and partially visible targets.

### 2.2. YOLO11n Baseline Model

The YOLO series represents a typical one-stage object detection framework, in which object category prediction and bounding-box regression are performed simultaneously during a single forward pass. Owing to this design, YOLO models generally offer high detection efficiency and strong potential for real-time applications [23]. YOLO11n follows the efficient detection paradigm of the YOLO family while further optimizing the feature extraction structure and multi-scale feature fusion strategy, achieving a favorable balance among detection accuracy, model size, and inference speed. In intelligent sheep farming, fixed surveillance video streams often need to be analyzed continuously. Therefore, a sheep detection model should not only provide effective target representation but also maintain low computational complexity, a compact parameter scale, and practical deployment efficiency [24,25]. For these reasons, the lightweight YOLO11n model was selected as the baseline in this study, and structural improvements were developed based on it.

**Figure 3:**
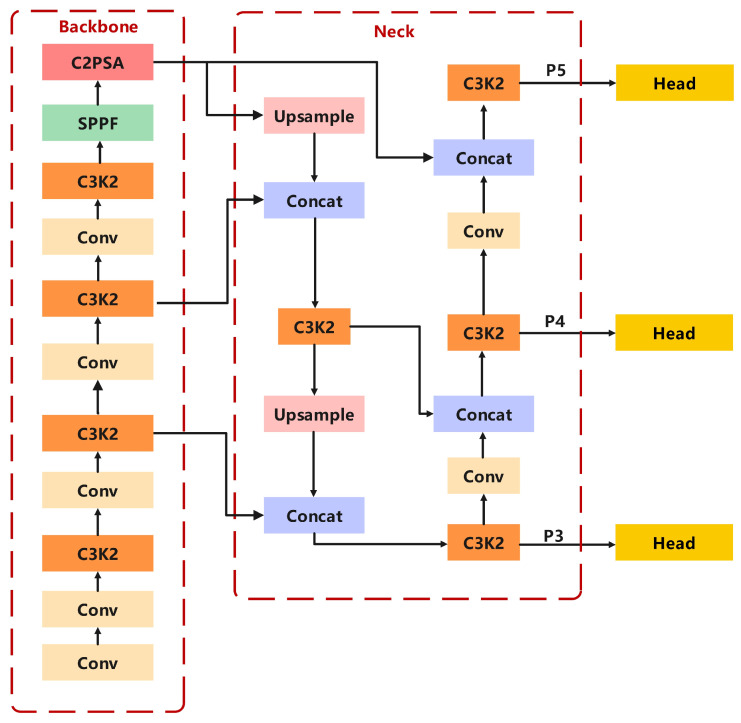
Schematic diagram of the YOLO11n baseline model.

In terms of overall architecture, YOLO11n mainly consists of three components: the backbone, the neck, and the detection head. The backbone is responsible for hierarchical image feature extraction. As the network depth increases, the spatial resolution of the feature maps gradually decreases, while their semantic representation capability becomes stronger. The neck performs multi-scale feature fusion to combine high-level semantic information with low-level spatial details. The detection head then uses the fused features to perform object classification and bounding-box regression. YOLO11n typically adopts three detection scales, namely P3/8, P4/16, and P5/32, to accommodate the recognition and localization of targets of different sizes. The overall architecture is illustrated in Figure 3.

### 2.3. Overall Framework of the Improved Model

**Figure 4:**
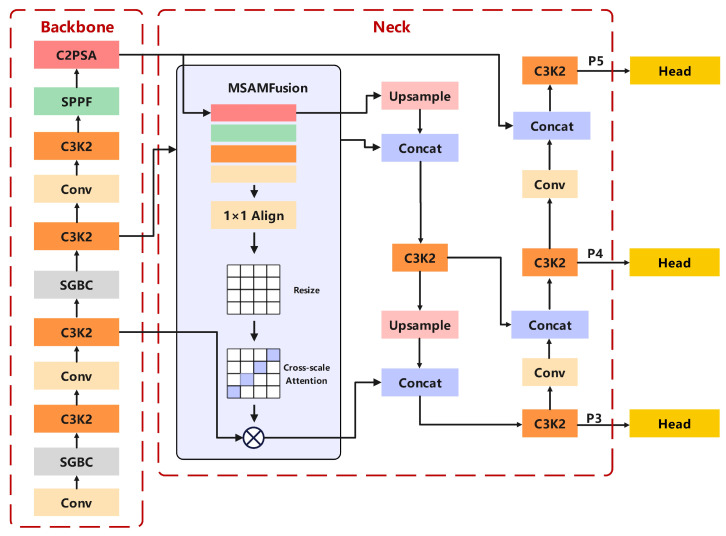
Overall architecture of GMS-YOLO11n.

In this study, an improved sheep detection model was developed for the challenging fixed-view farm conditions represented in the current dataset, using the lightweight YOLO11n architecture as the baseline. The complete model integrating SGBC and MSAMFusion was named GMS-YOLO11n. The overall network retains the basic Backbone–Neck–Head detection framework, and the original Detect head is kept unchanged. This design allows targeted structural optimization while preserving the original detection pipeline as much as possible and limiting the additional computational cost. In real farm environments, sheep targets are frequently affected by dense occlusion, scale variation, and complex background interference, which may lead to missed detections, false detections, and inaccurate bounding-box localization [26,27]. Meanwhile, the baseline model still has room for improvement in preserving local structural information and explicitly modeling cross-scale feature interactions. Therefore, YOLO11n was mainly improved from two aspects: shallow and shallow-to-intermediate structural enhancement, and collaborative multi-scale feature fusion. The overall architecture of the proposed model is shown in Figure 4.

First, a spatially gated bottleneck convolution module (SGBC) was introduced into both the shallow and shallow-to-intermediate feature extraction stages of the backbone. Specifically, the first SGBC module was used to replace the original downsampling convolution from the P1/2 stage to the P2/4 stage, whereas the second SGBC module was used to replace the original downsampling convolution from the P3/8 stage to the P4/16 stage. Under fixed surveillance views, structural cues such as sheep edges, contours, local textures, and contact regions between adjacent individuals may be weakened during successive convolution and downsampling operations. These cues can also be disturbed by background noise, including fences, ground textures, and shadows. Previous studies have shown that spatial details and boundary information contained in low-level features play an important role in object localization under complex scenarios [28]. Based on this consideration, SGBC selectively enhances local structural features through lightweight bottleneck convolution and an explicit spatial gating mechanism. This improves the representation of detailed information at key feature extraction stages and provides more discriminative structural features for subsequent multi-scale fusion.

Second, a multi-scale attention fusion module (MSAMFusion) was introduced at the entrance of the neck. This module receives four-scale features, namely P2/4, P3/8, P4/16, and P5/32, from the backbone. Through channel alignment, spatial alignment, and lightweight multi-scale fusion, features from different levels are first aggregated. On this basis, cross-scale attention interaction is performed with the high-level semantic feature P5 as the central feature, thereby promoting the collaborative representation of deep semantic information and shallow-to-intermediate detailed information. MSAMFusion further incorporates a channel-wise learnable gating mechanism at its output to regulate the contribution of the newly introduced fusion representation. This mechanism progressively integrates the enhanced information with the aligned high-level feature and produces the refined representation P5′.

Subsequently, P5′ is concatenated with the high-level feature output from the C2PSA module in the backbone, and the concatenated feature is integrated through a C3K2 module. The model then continues to follow a hierarchical fusion pathway composed of upsampling, feature concatenation, and C3K2 modules, ultimately forming three detection branches corresponding to P3, P4, and P5. While controlling the additional computational burden, this design was intended to improve feature representation under occlusion, scale variation, and reduced target–background contrast [29,30].

### 2.4. SGBC Local Structural Enhancement Module

**Figure 5:**
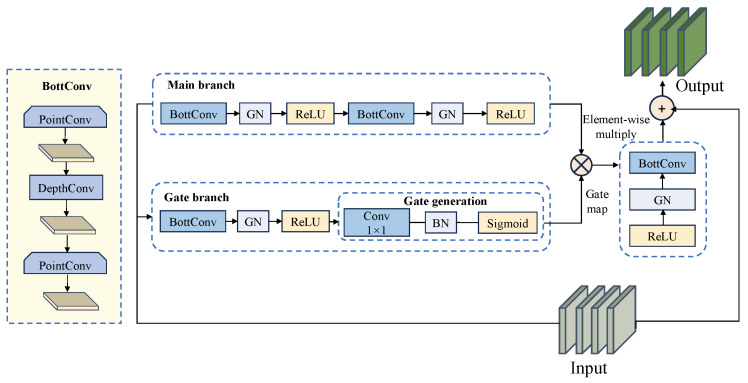
Structure of the SGBC local structural enhancement module.

To enhance the representation of structural information, including sheep edges, contours, local textures, and contact regions between adjacent individuals, a spatially gated bottleneck convolution module (SGBC) was introduced into the shallow and shallow-to-intermediate feature extraction stages of the YOLO11n backbone. The structure of SGBC is shown in Figure 5. Specifically, the first SGBC module was used to replace the downsampling convolution from the P1/2 stage to the P2/4 stage, whereas the second SGBC module was used to replace the downsampling convolution from the P3/8 stage to the P4/16 stage.

The proposed module was inspired by the gated bottleneck convolution (GBC) proposed by Liu et al. [31]. The original GBC reduces the number of parameters and computational complexity through BottConv and enhances the modeling of object morphology and complex backgrounds through gated feature interaction. However, unlike the original GBC, which mainly focuses on morphological and textural cue modeling in crack segmentation, the SGBC module in this study was adapted for sheep detection under challenging fixed-view farm conditions. By introducing an explicit spatial gating modulation mechanism, SGBC strengthens local structural features associated with sheep targets. It does not replace the basic convolutional structure throughout the entire backbone; instead, it makes local adjustments at key downsampling stages. This design provides more discriminative structural information for subsequent multi-scale feature fusion while controlling the additional computational cost.

Let the input feature be defined as follows:

(1) $$x\in R^{C_{1}\times H\times W}$$

where $C_{1},\;H$, and W denote the number of input channels, height, and width, respectively. Following the design concept of lightweight bottleneck convolution and gated feature interaction in GBC, BottConv was used as the basic computational unit of SGBC. BottConv adopts a bottleneck structure consisting of pointwise convolution, depthwise convolution, and pointwise convolution. Given the input feature x, BottConv can be expressed as:

(2) $$\mathrm{BottConv}\left(x\right)=PW_{2}(\mathrm{DW}(PW_{1}(x)))$$

where $PW_{1}\left(\cdot \right)$ and $PW_{2}\left(\cdot \right)$ denote 1×1 pointwise convolutions, and $\mathrm{DW}\left(\cdot \right)$ denotes depthwise convolution. This structure first performs channel mapping and compression through pointwise convolution, then extracts local spatial information using depthwise convolution, and finally restores the output channel dimension. In this way, BottConv maintains the ability to model local structural features with relatively low parameter and computational costs.

In SGBC, the input feature is first fed into the main branch to extract local structural responses. The main branch consists of two consecutive BottConv layers, normalization operations, and ReLU activation functions. Its output is denoted as $x_{1}$:

(3) $$x_{1}=\delta \left({GN}_{2}\left(\mathrm{BottCon}v_{2}\left(\delta \left({GN}_{1}\left(\mathrm{BottCon}v_{1}\left(x\right)\right)\right)\right)\right)\right)$$

where $\delta \left(\cdot \right)$ denotes the ReLU activation function, and $\mathrm{GN}\left(\cdot \right)$ denotes group normalization. This branch is mainly used to progressively refine the input shallow features and enhance the representation of sheep edges, local textures, and morphological structures.

Meanwhile, SGBC constructs a parallel gating branch to generate spatially selective weights. This branch first extracts gating-response features $x_{2}$ through BottConv:

(4) $$x_{2}=\delta \left({GN}_{3}\left(\mathrm{BottCon}v_{3}\left(x\right)\right)\right)$$

Then, $x_{2}$ is passed through a 1×1 convolution, batch normalization, and a Sigmoid activation function to generate a single-channel spatial gating map g:

(5) $$g=\sigma \left(\mathrm{BN}\left({\mathrm{Conv}}_{1\times 1}\left(x_{2}\right)\right)\right)$$

where $\sigma \left(\cdot \right)$ denotes the Sigmoid function. Through this design, the gating response is constrained to the range of $\left[0,1\right]$, allowing the module to adaptively assign weights to different spatial locations according to the input content. Compared with direct multiplicative modulation using parallel features, explicitly generating a single-channel spatial gating map helps strengthen feature responses in informative local regions and, to some extent, reduces interference from irrelevant areas such as background textures, shadows, and fences.

After obtaining the main-branch feature $x_{1}$ and the spatial gating map g, SGBC adaptively modulates the main-branch response through element-wise multiplication:

(6) $$m\left(x\right)=x_{1}⊙g$$

where ⊙ denotes element-wise multiplication. Because g is a single-channel spatial weight map, it is broadcast along the channel dimension to modulate $x_{1}$. This process enhances key structural responses, such as sheep contours, boundary regions, local textures, and contact areas between adjacent individuals, while suppressing relatively irrelevant background regions.

The gated feature $m\left(x\right)$ is then further refined by an additional BottConv layer to obtain the enhanced feature y:

(7) $$y=\delta \left({\mathrm{GN}}_{4}\left(\mathrm{BottCon}v_{4}\left(m\left(x\right)\right)\right)\right)$$

To preserve the original feature flow and improve training stability, SGBC introduces a residual shortcut mapping $S\left(x\right)$ at the output. The final output of the module is defined as:

(8) $$\mathrm{SGBC}\left(x\right)=y+S\left(x\right)$$

When the number of input and output channels is the same and the stride is 1, $S\left(x\right)$ is an identity mapping. When the number of channels differs or spatial downsampling is required, a 1×1 convolution with the corresponding stride, followed by normalization, is used to align the channel number and spatial size.

From the perspective of task adaptation, SGBC selectively enhances shallow structural information, including sheep edges, contours, and local textures, through lightweight bottleneck convolution and an explicit spatial gating mechanism. While limiting additional computational overhead, the module provides more discriminative low-level information for subsequent cross-scale feature fusion.

### 2.5. Design of the MSAMFusion Module

**Figure 6:**
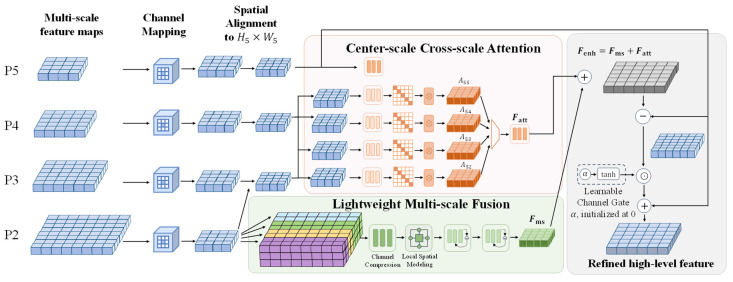
Structure of the MSAMFusion module.

To improve feature representation for sheep affected by partial occlusion, scale variation, and low visual contrast under the fixed-view conditions represented in the present dataset, a multi-scale attention fusion module, termed MSAMFusion, was introduced at the entrance of the YOLO11n neck. The module was inspired by the multi-scale self-attention mechanism proposed by Li et al. [32]. Whereas the original MSAM was designed for global contextual modeling in remote-sensing semantic segmentation, MSAMFusion adapts its cross-scale interaction principle to object detection. Specifically, the proposed module combines multi-scale feature aggregation, P5-centered cross-scale attention, and channel-wise gated feature injection. Its structure is shown in Figure 6.

Let the four backbone feature maps supplied to MSAMFusion be denoted as

(9) $$P_{i}\in R^{B\times C_{i}\times H_{i}\times W_{i}},\;i\in \{2,3,4,5\}$$

where B, $C_{i}$, $H_{i}$, and $W_{i}$ denote the batch size, number of channels, feature-map height, and feature-map width at the i-th scale, respectively.

The channel dimensions specified in the YAML configuration are nominal values before width scaling. The instantiated YOLO11n-n configuration applies a width multiplier of 0.25. Therefore, the nominal backbone channels of 256, 512, 512, and 1024 are instantiated as 64, 128, 128, and 256 channels, respectively. For an input resolution of 640 × 640 pixels, MSAMFusion receives the following feature tensors from backbone layers 2, 4, 6, and 8:

(10) $$P_{2}\in R^{B\times 64\times 160\times 160}$$

(11) $$P_{3}\in R^{B\times 128\times 80\times 80}$$

(12) $$P_{4}\in R^{B\times 128\times 40\times 40}$$

(13) $$P_{5}\in R^{B\times 256\times 20\times 20}$$

Each feature map is independently projected to a common embedding dimension of c = 256 using a 1 × 1 Conv-BN-SiLU block:

(14) $${\hat{P}}_{i}=\phi_{i}\left(P_{i}\right),\;i\in \{2,3,4,5\}$$

where $\phi_{i}\left(\cdot \right)$ denotes the channel-mapping operation. The corresponding channel transformations are 64 → 256, 128 → 256, 128 → 256, and 256 → 256.

The spatial resolution of ${\hat{P}}_{5}$ is used as the reference. The higher-resolution features are resized to 20 × 20 using nearest-neighbor interpolation, whereas ${\hat{P}}_{5}$ retains its original spatial resolution:

(15) $${\tilde{P}}_{i}={Resize}_{20\times 20}\left({\hat{P}}_{i}\right),\;i\in \{2,3,4\}$$

(16) $${\tilde{P}}_{5}={\hat{P}}_{5}$$

After channel and spatial alignment, all four feature maps have the same tensor shape:

(17) $${\tilde{P}}_{i}\in R^{B\times 256\times 20\times 20},\;i\in \{2,3,4,5\}$$

After channel and spatial alignment, the four aligned feature maps were aggregated by parameter-free element-wise averaging:

(18) $$F_{ms}=\frac{{\tilde{P}}_{5}+{\tilde{P}}_{4}+{\tilde{P}}_{3}+{\tilde{P}}_{2}}{4}.$$

The resulting multi-scale representation retained the following tensor shape:

(19) $$F_{ms}\in R^{B\times 256\times 20\times 20}$$

This parameter-free operation aggregates information from the four aligned feature levels without introducing additional trainable parameters.

In parallel, a P5-centered cross-scale attention branch is constructed. The custom model parser applies the YOLO11n-n width multiplier to both the nominal MSAMFusion embedding dimension and the nominal attention dimension. Consequently, the nominal embedding dimension of 1024 is instantiated as c = 256, whereas the nominal attention dimension of 256 is instantiated as d = 64.

Because ${\tilde{P}}_{5}$ contains the highest-level semantic information among the four aligned features, it is used to generate the query. The four aligned feature maps are used to generate the corresponding keys and values:

(20) $$Q=\psi_{Q}\left({\tilde{P}}_{5}\right)$$

(21) $$K_{j}=\psi_{K}\left({\tilde{P}}_{j}\right),\;V_{j}=\psi_{V}\left({\tilde{P}}_{j}\right),\;j\in \{5,4,3,2\}$$

where $\psi_{Q}\left(\cdot \right)$, $\psi_{K}\left(\cdot \right)$, and $\psi_{V}\left(\cdot \right)$ denote the query, key, and value projection operations, respectively. The key and value projection parameters are shared across the four feature scales. Each projection maps the aligned 256-channel feature to a 64-dimensional embedding:

(22) $$Q,K_{j},V_{j}\in R^{B\times 64\times 20\times 20}$$

The attention embedding is divided into h = 4 heads. The dimension of each head is therefore

(23) $$d_{h}=\frac{d}{h}=\frac{64}{4}=16$$

After flattening the spatial dimensions, the query, key, and value tensors are reshaped into a multi-head representation with N = 20 × 20 = 400 spatial tokens. Scaled dot-product attention is then calculated between the central P5 query and each aligned feature scale:

(24) $$A_{5j}=Softmax\left(\frac{QK_{j}^{T}}{\sqrt{d_{h}}}\right)V_{j},\;j\in \{5,4,3,2\}$$

This operation produces four 64-channel attention outputs, denoted as $A_{55}$, $A_{54}$, $A_{53}$, and $A_{52}$. Here, $A_{55}$ represents attention interaction within the central scale, whereas $A_{54}$, $A_{53}$, and $A_{52}$ represent complementary information obtained from the remaining feature levels.

The four 64-channel attention outputs were aggregated by parameter-free element-wise averaging and subsequently mapped to 256 channels using a single 1×1 Conv-BN output projection:

(25) $$F_{att}=\psi_{O}\left(\frac{A_{55}+A_{54}+A_{53}+A_{52}}{4}\right)$$

where $\psi_{O}\left(\cdot \right)$ denotes the 64→256 output projection. The resulting cross-scale attention representation retained the following tensor shape:

(26) $$F_{att}\in R^{B\times 256\times 20\times 20}$$

The multi-scale and cross-scale attention representations were combined by element-wise addition:

(27) $$F_{enh}=F_{ms}+F_{att}$$

where

(28) $$F_{\mathrm{enh}}\in R^{B\times 256\times 20\times 20}$$

Instead of directly replacing the aligned high-level feature, a channel-wise learnable gate is used to regulate the contribution of the enhanced representation. The learnable gating parameter is defined as:

(29) $$\alpha \in R^{1\times 256\times 1\times 1}$$

and is initialized to zero. The final output of MSAMFusion is calculated as:

(30) $$P_{5}^{\prime }={\tilde{P}}_{5}+tanh\left(\alpha \right)⊙\left(F_{\mathrm{enh}}-{\tilde{P}}_{5}\right)$$

Because $tanh\left(0\right)=0$, the initial output of the gated-injection operation is identical to the aligned high-level feature ${\tilde{P}}_{5}$. During training, the channel-wise gate parameters are jointly optimized with the network, allowing the contribution of the enhanced cross-scale representation to be adjusted independently across channels.

The final MSAMFusion output retains the following tensor shape:

(31) $$P_{5}^{\prime }\in R^{B\times 256\times 20\times 20}$$

MSAMFusion therefore produces a 256-channel high-level representation by combining multi-scale feature aggregation, low-dimensional P5-centered cross-scale attention, and channel-wise gated feature injection. The output is subsequently supplied to the high-level feature-fusion pathway of the neck.

## 3. Results

### 3.1. Experimental Setup

All experiments were conducted on a Linux workstation equipped with an NVIDIA GeForce RTX 4090 GPU. Model training and evaluation were performed using PyTorch 2.3.0, CUDA 12.1, and Ultralytics 8.3.240. All input images were resized to 640 × 640 pixels, and the batch size was set to 16. Each model was trained for 150 epochs using stochastic gradient descent with an initial learning rate of 0.001, a momentum of 0.937, a weight decay of 0.0005, and a three-epoch warm-up period. Mosaic augmentation was disabled during the final 10 epochs. Random horizontal flipping, translation, scaling, and HSV augmentation were applied. The horizontal-flipping probability was 0.5, the translation and scaling factors were 0.1 and 0.5, respectively, and the hue, saturation, and value coefficients were 0.015, 0.7, and 0.4, respectively.

All baseline detectors were initialized using their corresponding COCO-pretrained checkpoints. For GMS-YOLO11n, parameters with matching layer names and tensor dimensions were transferred from the COCO-pretrained YOLO11n checkpoint. The newly introduced layers in SGBC and MSAMFusion were initialized using the default initialization settings of the framework, whereas the channel-wise gating parameter α was initialized to zero. The random seeds controlled stochastic operations during training and did not represent random initialization of the complete networks. All models used the same recording-session-level training, validation, and test partitions. For each model configuration and random seed, the checkpoint with the highest validation-set mAP50–95 was selected. The validation set was used only for performance monitoring and checkpoint selection. All model configurations and training hyperparameters were fixed before training, and the independent test set was used only for the final evaluation of the selected checkpoints.

The component-combination ablations, progressive internal ablations, and gate-control experiments were repeated using six matched random seeds (0–5). The dataset partition, initialization procedure, training settings, checkpoint-selection criterion, and evaluation procedure were kept consistent across all repeated runs. The results are reported as the mean ± sample standard deviation. The cross-model comparison and computational-efficiency evaluation were conducted using the seed-0 runs. For the cross-model comparison, the recording-session-level dataset partition, input resolution, batch size, optimizer, initial learning rate, training duration, data-augmentation settings, checkpoint-selection criterion, and independent test procedure were predefined and kept consistent across all evaluated models. No model-specific hyperparameter tuning was performed. Accordingly, the reported cross-model results represent controlled comparisons under a common predefined experimental protocol. Computational efficiency was measured independently for each model on an NVIDIA GeForce RTX 4090 GPU using FP32 precision, an input resolution of 640 × 640 pixels, and a batch size of 16 after warm-up. The reported processing times represent the average time per image. Peak GPU memory was recorded as the maximum GPU memory allocated by PyTorch during inference. The complete GMS-YOLO11n YAML configuration, nominal and instantiated channel dimensions, layer-wise architecture and parameter summary, MSAMFusion tensor shapes, module-wise parameter counts and GFLOPs, and framework-generated model summary are provided in the Supplementary Materials.

### 3.2. Performance Evaluation Metrics

Detection performance was assessed using precision, recall, F1-score, mAP@0.5 [33], and mAP@0.5:0.95 [34]. Model complexity was characterized by the number of parameters (Params) and giga floating-point operations (GFLOPs). Precision, recall, and F1-score were calculated as follows [35]:

(32) $$P=\frac{TP}{TP+FP}$$

(33) $$R=\frac{TP}{TP+FN}$$

(34) $$F_{1}=\frac{2\times P\times R}{P+R}$$

where TP, FP, and FN denote the numbers of true-positive, false-positive, and false-negative detections, respectively. Precision reflects the proportion of correct detections among all predicted sheep instances, whereas recall reflects the proportion of annotated sheep instances detected by the model. The F1-score summarizes the balance between precision and recall.

Average precision was obtained from the area under the precision–recall curve. Mean average precision was calculated as:

(35) $$mAP=\frac{1}{C}\sum_{i=1}^{C}AP_{i}$$

where $AP_{i}$ denotes the average precision for the i-th class and C is the number of detection classes [36]. Because sheep was the only class in this study, C = 1. mAP@0.5 denotes AP at an intersection-over-union threshold of 0.50, whereas mAP@0.5:0.95 denotes AP averaged over IoU thresholds from 0.50 to 0.95 at intervals of 0.05. The latter applies stricter localization criteria and therefore provides a more comprehensive measure of bounding-box accuracy. For brevity, mAP@0.5 and mAP@0.5:0.95 are referred to as mAP50 and mAP50–95, respectively, in the following sections.

Computational efficiency was evaluated using preprocessing time, inference time, postprocessing time, total latency, throughput, and peak GPU memory. All processing times were reported in milliseconds per image. Total latency was calculated as the sum of preprocessing, inference, and postprocessing times. Throughput was derived as 1000 divided by total latency and was reported in images/s. Peak GPU memory was defined as the maximum memory allocated by PyTorch during inference and was reported in GiB.

### 3.3. Training Convergence Analysis

**Figure 7:**
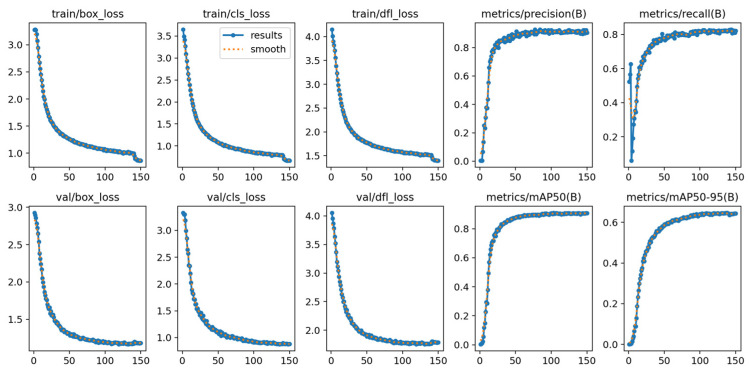
Training loss and detection performance curves of GMS-YOLO11n over 150 epochs.

Figure 7 shows the training and validation curves of GMS-YOLO11n over 150 epochs. The box-regression, classification, and distribution focal losses decreased rapidly during the early training stage and then gradually stabilized. The corresponding validation losses followed similar trends, with no pronounced divergence during the later epochs. Precision, recall, mAP50, and mAP50–95 increased progressively and reached relatively stable levels before the end of training. These results indicate that the selected 150-epoch schedule was sufficient for model convergence and suitable for the subsequent ablation experiments and model comparisons.

### 3.4. Ablation Experiment

To investigate the individual and combined effects of SGBC and MSAMFusion on sheep detection performance, YOLO11n was used as the baseline, and three variants were constructed: YOLO11n + SGBC, YOLO11n + MSAMFusion, and GMS-YOLO11n. Each configuration was independently trained using six matched random seeds under the experimental settings described in Section 3.1. Precision, recall, F1-score, mAP50, and mAP50–95 were used for performance evaluation. The results are presented in Table 2 as the mean ± standard deviation.

As shown in Table 2, the YOLO11n baseline achieved mean precision, recall, F1-score, mAP50, and mAP50–95 values of 92.57%, 86.65%, 89.51%, 93.77%, and 69.40%, respectively. After the introduction of SGBC, recall, F1-score, mAP50, and mAP50–95 increased by 1.92, 1.13, 1.33, and 4.68 percentage points, respectively. The greater improvement in mAP50–95 indicates that SGBC contributed more substantially to detection performance under stricter IoU thresholds. By strengthening local structural information, including sheep contours, boundary regions, and contact areas between adjacent individuals, SGBC improved feature representation for bounding-box regression and localization.

When MSAMFusion was introduced alone, precision, recall, F1-score, mAP50, and mAP50–95 reached 93.42%, 88.46%, 90.85%, 95.42%, and 71.90%, respectively, corresponding to improvements of 0.85, 1.81, 1.34, 1.65, and 2.50 percentage points over YOLO11n. These results indicate that multi-scale feature alignment and cross-scale interaction improved both detection completeness and localization performance by integrating high-level semantic information with shallow-to-intermediate details.

**Figure 8:**
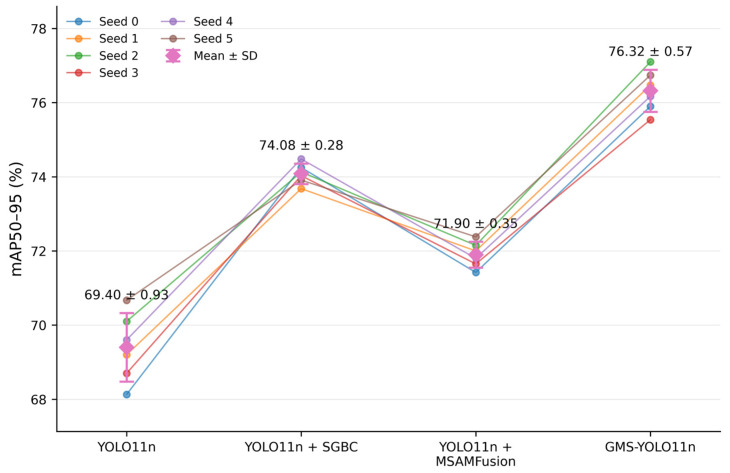
Seed-wise mAP50–95 results across six matched random seeds.

With the simultaneous introduction of SGBC and MSAMFusion, GMS-YOLO11n achieved the highest mean values across all five evaluation metrics, with precision, recall, F1-score, mAP50, and mAP50–95 values of 93.62%, 91.03%, 92.30%, 96.53%, and 76.32%, respectively. Compared with YOLO11n, the corresponding improvements were 1.05, 4.38, 2.79, 2.76, and 6.92 percentage points. The two-sided 95% confidence intervals for mAP50–95 were 68.42–70.38% for YOLO11n and 75.72–76.92% for GMS-YOLO11n. The seed-wise results in Figure 8 further show that the performance advantage of the complete model was maintained across the six matched random seeds. These results indicate that SGBC and MSAMFusion contributed at different stages of feature processing: SGBC refined local structural information in the backbone, whereas MSAMFusion subsequently integrated the multi-scale feature hierarchy in the neck. The progressive internal ablation and gate-control results in Section 3.5 and Section 3.6 further support the effectiveness of the complete configuration.

### 3.5. Ablation Experiment on the Internal Components of MSAMFusion

To examine the contribution of the internal components of MSAMFusion, progressive ablation experiments were conducted on the YOLO11n + SGBC configuration using six matched random seeds. B1 included only feature alignment and multi-scale aggregation, whereas B2 additionally incorporated the P5-centered cross-scale attention branch. B3 further introduced the channel-wise learnable gate and represents the complete GMS-YOLO11n model. In B2, the enhanced representation was forwarded directly to the subsequent neck. In B3, it was integrated with the aligned high-level feature through the learnable gate. B2 and B3 used the same fusion branches, downstream architecture, initialization protocol, and training settings, differing only in the output feature-injection strategy. The results are reported as the mean ± sample standard deviation in Table 3.

Relative to YOLO11n + SGBC, B1 increased precision by 0.72 percentage points but reduced recall and mAP50–95 by 1.76 and 4.80 percentage points, respectively. The addition of cross-scale attention in B2 did not reverse this trend, with recall and mAP50–95 decreasing to 85.84% and 68.60%. These results show that constructing a multi-scale enhanced representation, either with or without the attention branch, was not sufficient to obtain consistent performance gains when the resulting feature was forwarded without channel-wise regulation. B3 achieved the highest mean values for all five evaluation metrics. Compared with B2, precision, recall, F1-score, mAP50, and mAP50–95 increased by 0.93, 5.19, 3.17, 3.54, and 7.72 percentage points, respectively. The 95% confidence intervals for mAP50–95 were 67.98–69.22% for B2 and 75.72–76.92% for B3. Because B2 and B3 differed only in the output feature-injection strategy, these results indicate that the manner in which the enhanced representation was incorporated into the high-level feature affected the performance of MSAMFusion. The zero-initialized gate preserved the aligned high-level feature at the beginning of training and allowed the contribution of the enhanced representation to be adjusted progressively during optimization.

### 3.6. Gate-Control Ablation of MSAMFusion

To evaluate the effects of different feature-injection strategies and gate initializations, controlled ablation experiments were conducted using B2 as the reference configuration. All variants retained the same multi-scale fusion branch, cross-scale attention branch, downstream network structure, and training settings, and differed only in the output-injection strategy or gate configuration. The learnable-gate variants were initialized with $\alpha_{0}=0.5$, $\alpha_{0}=1.0$, and $\alpha_{0}=0$, respectively, with the effective gate defined as $g=tanh\left(\alpha \right)$. Fixed-0.10 used a constant gate coefficient of g=0.10, whereas the residual-addition variant directly combined the aligned high-level feature with the enhanced representation through element-wise addition. Each configuration was evaluated using six matched random seeds, and the results are reported as the mean ± standard deviation in Table 4. The learnable-gate variants initialized with $\alpha_{0}=0.5$ and $\alpha_{0}=1.0$ achieved mAP50–95 values of 69.14% ± 0.55% and 69.15% ± 0.51%, respectively, while Fixed-0.10 and residual addition achieved 69.72% ± 0.54% and 69.54% ± 0.60%. These results were only moderately higher than that of B2, which achieved 68.60% ± 0.59%.

**Figure 9:**
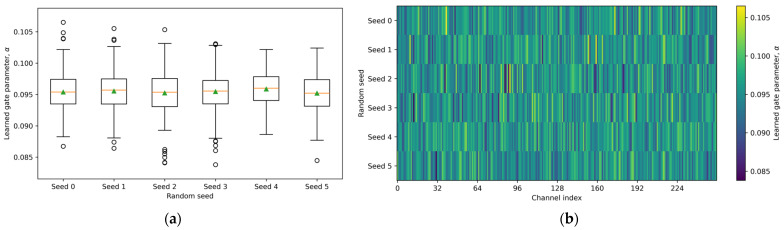
Distributions of the learned channel-wise gate parameters α across six matched random seeds: (**a**) box plots of the channel-wise parameter distributions for each seed; and (**b**) heat map of the learned parameter values across channels and seeds.

B3, initialized with $\alpha_{0}=0$, achieved the highest mean values across all five evaluation metrics, with precision, recall, F1-score, mAP50, and mAP50-95 values of 93.62% ± 0.12%, 91.03% ± 1.37%, 92.30% ± 0.75%, 96.53% ± 0.49%, and 76.32% ± 0.57%, respectively. Compared with B2, the corresponding improvements were 0.93, 5.19, 3.17, 3.54, and 7.72 percentage points. These findings indicate that direct residual addition, fixed gating, and larger gate initializations did not provide performance comparable to that of the zero-initialized learnable gate. When $\alpha_{0}=0$, the module initially preserves the aligned high-level feature because $g=tanh\left(\alpha \right)=0$, after which the contribution of the enhanced cross-scale representation is progressively adjusted during training. The learned channel-wise parameters α were mainly concentrated around 0.095 and showed similar overall distributions across the six matched random seeds, with modest channel-wise variation, as shown in Figure 9.

### 3.7. Performance Analysis of Different Detection Models

Table 5 presents the seed-0 results of the seven evaluated detectors on the independent test set. Each baseline model was implemented using its corresponding architecture definition in Ultralytics 8.3.240 and initialized with the corresponding COCO-pretrained checkpoint. For GMS-YOLO11n, parameters with matching layer names and tensor dimensions were transferred from the same COCO-pretrained YOLO11n checkpoint used for the baseline. All models were trained and evaluated using the same recording-session-level dataset partition, input resolution, batch size, optimizer, initial learning rate, training duration, data-augmentation settings, checkpoint-selection criterion, and independent test procedure, while retaining their architecture-specific components. No model-specific hyperparameter tuning was performed. Therefore, Table 5 presents a controlled comparison under a common predefined experimental protocol rather than the individually optimized performance of each architecture.

Among the baseline models, YOLO11n achieved the highest precision, recall, F1-score, and mAP50, whereas YOLOv8n achieved the highest mAP50–95. GMS-YOLO11n achieved precision, recall, F1-score, mAP50, and mAP50–95 values of 93.50%, 90.07%, 91.75%, 95.98%, and 75.94%, respectively. Compared with YOLO11n, these metrics increased by 0.72, 3.39, 2.12, 2.47, and 7.57 percentage points, respectively. The proposed model contained 3.10 M parameters and required 7.90 GFLOPs, corresponding to increases of 0.52 M parameters and 1.59 GFLOPs over YOLO11n. At an input resolution of 640 × 640 pixels, SGBC-1 and SGBC-2 contained 1874 and 34,626 parameters and required 0.096 and 0.111 GFLOPs, respectively. The two SGBC modules therefore contained 36,500 parameters and required 0.207 GFLOPs in total. MSAMFusion contained 216,192 parameters and required 1.710 GFLOPs. Detailed module-wise complexity statistics are provided in Table S5 of the Supplementary Materials.

As shown in Table 6, YOLOv10n achieved the highest throughput, whereas RT-DETR-L had the highest peak GPU memory usage. GMS-YOLO11n achieved a total latency of 3.50 ms per image, a throughput of 285.71 images/s, and a peak GPU memory usage of 0.69 GiB. Compared with YOLO11n, the proposed model increased total latency by 0.42 ms per image and peak GPU memory usage by 0.12 GiB. These results indicate that the proposed modifications introduced moderate computational and memory overhead while maintaining high batch-processing throughput under the evaluated hardware setting.

### 3.8. Detection Performance Analysis Under Different Scenario Subsets

To further characterize detection performance under the visual conditions represented in the current dataset, a stratified evaluation was conducted using the image-level scene attributes described in Section 2.1. Illumination condition and occlusion degree were considered as two independent evaluation dimensions. GMS-YOLO11n was compared with the YOLO11n baseline using the validation-selected checkpoints from the predefined seed-0 runs. The test subsets were used only for stratified performance evaluation. Based on illumination condition, the test images were divided into daytime natural-light and nighttime low-light subsets. Based on occlusion degree, they were divided into subsets without obvious occlusion and with obvious occlusion. Because illumination and occlusion were independently assigned image-level attributes, an image could simultaneously belong to one illumination subset and one occlusion subset. The results for the two evaluation dimensions were therefore analyzed separately, as shown in Table 7.

As shown in Table 7, GMS-YOLO11n outperformed YOLO11n across all evaluated scenario subsets. The largest improvement was observed under nighttime low-light conditions, where recall and mAP50–95 increased by 6.63 and 9.83 percentage points, respectively. In scenes with obvious occlusion, recall and mAP50–95 increased by 5.12 and 7.62 percentage points, respectively. These results indicate that the proposed model improved detection completeness and bounding-box localization particularly under reduced illumination and partial target visibility. Nevertheless, performance remained lower in the obvious-occlusion subset than in the subset without obvious occlusion, indicating that severe overlap, fence interference, and limited visible target regions remain challenging.

### 3.9. Visualization Analysis of Detection Results Under Representative Challenging Conditions

**Figure 10:**
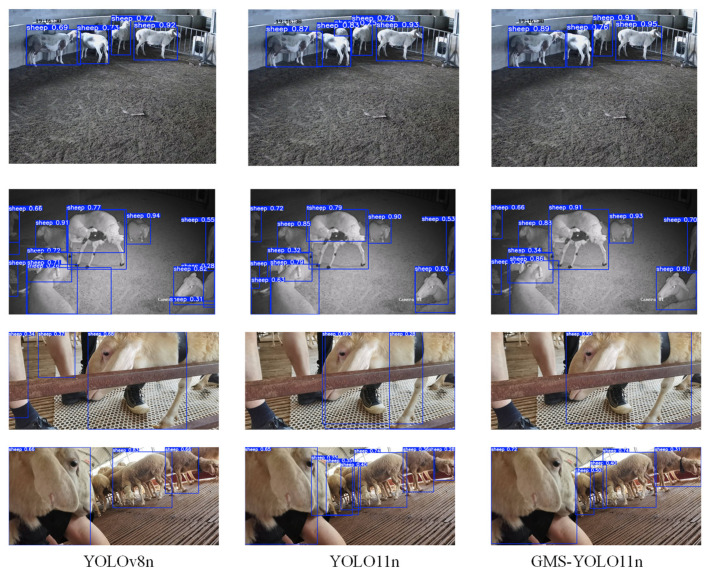
Qualitative detection results under representative challenging conditions.

Figure 10 compares the detection results of YOLOv8n, YOLO11n, and GMS-YOLO11n under four representative conditions in the current dataset. The three columns correspond to YOLOv8n, YOLO11n, and GMS-YOLO11n, respectively, while the rows show distant small-scale targets, nighttime low-light scenes, fence occlusion with human interference, and locally crowded sheep. In the daytime example, all three models detected the distant sheep, although GMS-YOLO11n retained slightly higher confidence scores for several small targets. Under nighttime low-light conditions, GMS-YOLO11n maintained detections for the central sheep and several partially visible animals near the image boundaries. In the fence-occlusion example, YOLOv8n produced several additional responses near the person and surrounding structures, whereas fewer such responses were observed for GMS-YOLO11n. In the crowded scene, GMS-YOLO11n detected both partially visible foreground sheep and smaller background sheep, with less overlap among adjacent predicted boxes than the two baseline models. These examples illustrate the behavior of the three models under the visual conditions represented in the current dataset and are consistent with the quantitative results reported in Section 3.7 and Section 3.8.

## 4. Discussion

Sheep detection under fixed-view farm monitoring is challenged by mutual occlusion, target-scale variation, illumination changes, and background interference. Across six matched-seed runs, GMS-YOLO11n improved precision, recall, F1-score, mAP50, and mAP50–95 by 1.05, 4.38, 2.79, 2.76, and 6.92 percentage points, respectively, compared with YOLO11n. The larger gains in recall and mAP50–95 suggest fewer missed detections and improved localization under stricter IoU criteria. The ablation results indicate that SGBC and MSAMFusion operate at different stages of feature processing. SGBC strengthens local structural information in the backbone, whereas MSAMFusion integrates multi-scale information with high-level semantic features in the neck. The progressive ablation and gate-control experiments further showed that the zero-initialized channel-wise learnable gate was more effective than direct feature forwarding, fixed gating, residual addition, or larger gate initializations.

The scenario-stratified evaluation showed that the performance gains were most pronounced under nighttime low-light and obvious-occlusion conditions. The larger improvements in recall and mAP50–95 suggest that the proposed feature-enhancement and fusion mechanisms were particularly beneficial for reducing missed detections and improving localization when target contrast and visible target extent were reduced. These results are consistent with improved detection completeness and localization in images affected by reduced illumination, mutual occlusion, fence interference, and partial visibility. The accuracy gains were accompanied by moderate computational and memory overhead. Relative to YOLO11n, total latency increased from 3.08 to 3.50 ms per image, throughput decreased from 324.68 to 285.71 images/s, and peak GPU memory increased from 0.57 to 0.69 GiB. Nevertheless, GMS-YOLO11n retained a compact model size of 3.10 M parameters and maintained high batch-processing throughput on the evaluated RTX 4090 platform. These measurements are hardware-specific and should not be interpreted as direct evidence of performance on resource-constrained edge devices.

This study was limited to 63 Small-tailed Han sheep from one breeding base and one primary fixed-camera configuration. The reported results therefore represent within-domain performance and do not establish generalization across farms, sheep breeds, flock compositions, imaging devices, camera viewpoints, or pen layouts. Although the recording-session-level partition prevented frames from the same continuous recording session from being distributed across the training, validation, and test sets, the test set was derived from only one 24 h recording session. Therefore, the reported test performance may be affected by the illumination conditions, flock distribution, occlusion patterns, and background characteristics represented in that particular session and does not quantify performance variability across multiple independent test sessions. Future work will include evaluation using multiple held-out recording sessions, followed by external validation across different farms, breeds, devices, viewpoints, and pen layouts, as well as deployment-oriented evaluation on resource-constrained hardware.

## 5. Conclusions

This study developed GMS-YOLO11n for sheep detection under the fixed-view farm conditions represented in the current dataset. The model incorporates SGBC to strengthen local structural information in the backbone and MSAMFusion to integrate multi-scale information with high-level semantic features in the neck. Across six matched random-seed runs, GMS-YOLO11n achieved mean precision, recall, F1-score, mAP50, and mAP50–95 values of 93.62%, 91.03%, 92.30%, 96.53%, and 76.32%, respectively. Compared with the COCO-pretrained YOLO11n baseline, recall and mAP50–95 increased by 4.38 and 6.92 percentage points, respectively. The scenario-stratified evaluation also showed improved performance under the nighttime low-light and obvious-occlusion conditions represented in the dataset. With 3.10 M parameters and 7.90 GFLOPs, the proposed model retained a compact architecture while improving detection performance. However, the evaluation was limited to 63 Small-tailed Han sheep from one breeding base and one primary fixed-camera configuration, and the test set was derived from only one 24 h recording session. Evaluation across multiple independent recording sessions and external datasets collected from different farms, breeds, devices, viewpoints, and pen layouts is therefore required before broader application.

## Figures and Tables

**Table 1:** Recording-session-level allocation and annotation composition of the experimental dataset.

Dataset	Recording Sessions	Raw Recording Duration/h	Images	Annotated Instances	Size	Proportion
Training	4	96	2824	6463	2560 × 1920	80%
Validation	1	24	353	848	2560 × 1920	10%
Test	1	24	354	856	2560 × 1920	10%
Total	6	144	3531	8167	2560 × 1920	100%

**Table 2:** Performance comparison of different combinations of improved components.

Model	Precision/%	Recall/%	F1-Score/%	mAP50/%	mAP50–95/%
YOLO11n	92.57 ± 0.90	86.65 ± 0.21	89.51 ± 0.53	93.77 ± 0.29	69.40 ± 0.93
YOLO11n + SGBC	92.83 ± 1.05	88.57 ± 1.66	90.64 ± 0.40	95.10 ± 0.08	74.08 ± 0.28
YOLO11n + MSAMFusion	93.42 ± 1.98	88.46 ± 1.62	90.85 ± 0.33	95.42 ± 0.50	71.90 ± 0.35
GMS-YOLO11n	93.62 ± 0.12	91.03 ± 1.37	92.30 ± 0.75	96.53 ± 0.49	76.32 ± 0.57

**Table 3:** Six-seed progressive ablation results for the internal components of MSAMFusion.

Model	Multi-Scale Fusion	Cross-Scale Attention	Learnable Gate	Precision/%	Recall/%	F1-Score/%	mAP50/%	mAP50–95/%
YOLO11n + SGBC	×	×	×	92.83 ± 1.05	88.57 ± 1.66	90.64 ± 0.40	95.10 ± 0.08	74.08 ± 0.28
B1	✓	×	×	93.55 ± 0.89	86.81 ± 1.30	90.05 ± 0.39	93.97 ± 0.66	69.28 ± 0.56
B2	✓	✓	×	92.69 ± 1.57	85.84 ± 0.94	89.13 ± 0.67	92.99 ± 0.05	68.60 ± 0.59
B3 (GMS-YOLO11n)	✓	✓	✓	93.62 ± 0.12	91.03 ± 1.37	92.30 ± 0.75	96.53 ± 0.49	76.32 ± 0.57

**Table 4:** Six-seed gate-control ablation results for different feature-injection strategies in MSAMFusion.

Variant	Learnable Gate	α Initialization	Precision (%)	Recall (%)	F1-Score (%)	mAP50 (%)	mAP50–95 (%)
B2	×	—	92.69 ± 1.57	85.84 ± 0.94	89.13 ± 0.67	92.99 ± 0.05	68.60 ± 0.59
Learnable-0.5	√	0.5	93.32 ± 1.20	85.91 ± 1.00	89.42 ± 0.65	93.71 ± 0.45	69.14 ± 0.55
Learnable-1.0	√	1.0	92.83 ± 1.25	85.84 ± 1.00	89.11 ± 0.65	93.14 ± 0.45	69.15 ± 0.51
Fixed-0.10	×	—	93.32 ± 1.10	86.07 ± 1.05	89.51 ± 0.65	94.25 ± 0.50	69.72 ± 0.54
Residual addition	×	—	93.11 ± 1.10	85.91 ± 1.10	89.32 ± 0.70	94.04 ± 0.55	69.54 ± 0.60
B3	√	0	93.62 ± 0.12	91.03 ± 1.37	92.30 ± 0.75	96.53 ± 0.49	76.32 ± 0.57

**Table 5:** Seed-0 performance and complexity comparison of different detection models.

Model	Initialization	Params (M)	GFLOPs	Precision (%)	Recall (%)	F1-Score (%)	mAP50 (%)	mAP50–95 (%)
RT-DETR-L	COCO pretrained	31.99	103.43	87.30	83.85	85.54	91.20	68.60
YOLOv5n	COCO pretrained	2.50	7.10	88.72	83.94	86.26	90.35	67.18
YOLOv8n	COCO pretrained	3.01	8.09	90.46	85.72	88.03	92.18	69.42
YOLOv9t	COCO pretrained	1.97	7.60	87.14	84.66	85.88	91.74	69.12
YOLOv10n	COCO pretrained	2.27	6.53	84.65	80.28	82.41	87.95	64.83
YOLO11n	COCO pretrained	2.58	6.31	92.78	86.68	89.63	93.51	68.37
GMS-YOLO11n	YOLO11n pretrained transfer	3.10	7.90	93.50	90.07	91.75	95.98	75.94

**Table 6:** Seed-0 computational-efficiency comparison of different detection models.

Model	Preprocess/ms	Inference/ms	Postprocess/ms	Total Latency/ms	Throughput/ (Images·s^−1^)	Peak GPU Memory/GiB
RT-DETR-L	0.20	4.03	0.17	4.40	227.27	1.35
YOLOv5n	0.17	1.20	0.81	2.18	458.72	0.43
YOLOv8n	0.19	1.35	0.94	2.48	403.23	0.49
YOLOv9t	0.13	1.53	0.34	2.00	500.00	0.54
YOLOv10n	0.19	1.21	0.05	1.45	689.66	0.64
YOLO11n	0.20	1.66	1.22	3.08	324.68	0.57
GMS-YOLO11n	0.20	2.08	1.22	3.50	285.71	0.69

**Table 7:** Detection performance comparison between YOLO11n and GMS-YOLO11n under different scenario subsets.

Scenario Type	Scenario Subset	Images	Instances	Model	Precision/%	Recall/%	F1-Score/%	mAP50/%	mAP50–95/%
Illumination condition	Daytime natural light	255	421	YOLO11n	94.48	94.50	94.49	98.37	77.59
				GMS-YOLO11n	95.47	96.90	96.18	98.63	81.80
	Nighttime low light	99	435	YOLO11n	87.50	78.15	82.56	87.69	59.38
				GMS-YOLO11n	89.92	84.78	87.28	92.83	69.21
Occlusion degree	Without obvious occlusion	121	387	YOLO11n	95.23	93.02	94.12	97.81	77.06
				GMS-YOLO11n	96.11	95.85	95.98	98.48	83.22
	With obvious occlusion	233	469	YOLO11n	88.76	82.09	85.30	89.80	61.61
				GMS-YOLO11n	90.69	87.21	88.91	93.99	69.23

## Data Availability

The data supporting the findings of this study are available from the corresponding author upon reasonable request, subject to project approval and applicable confidentiality requirements. The complete dataset is not publicly available at present because it was developed as part of an ongoing research project, is still being expanded and curated, and is subject to project-level confidentiality obligations.

## Supplementary Materials

The following supporting information can be downloaded at: https://www.mdpi.com/article/10.3390/ani16152351/s1, Table S1: Organization of the Supplementary Materials; Table S2: Nominal and instantiated channel dimensions under the YOLO11n n-scale configuration; Code Block S1: Complete GMS-YOLO11n YAML configuration; Table S3: Instantiated GMS-YOLO11n architecture and layer-wise parameter counts for a 640 × 640 input; Table S4: Tensor shapes at the inputs, intermediate operations, and output of MSAMFusion; Table S5: Module-wise parameter counts and GFLOPs of GMS-YOLO11n for a 640 × 640 input; Figure S1: Framework-generated layer summary of the instantiated GMS-YOLO11n model.
