# Supplementary material for: GMS-YOLO11n: A Sheep Detection Model for Challenging Fixed-View Farm Conditions Integrating Spatially Gated Structural Enhancement and Multi-Scale Attention
Source: Animals (Basel). 2026 Aug 1;16(15):2351. doi: 10.3390/ani16152351 (PMC13463468; doi:10.3390/ani16152351)
Supplement: Supplementary file 1 [file animals-16-02351-s001.zip › animals-4418743-supplementary.pdf]

# Supplementary Materials

# GMS-YOLO11n: A Sheep Detection Model for Challenging Fixed-View Farm Conditions Integrating Spatially Gated Structural Enhancement and Multi-Scale Attention

## Manuscript ID: animals-4418743

**Wenbo Yu, Ruoya Xie, Yongqi Liu, Zhi Xue, Zhengpeng Yang, and Wenqiang Han**
**Correspondence: xie2014030130@163.com**

## Table S1. Organization of the Supplementary Materials

| Item | Contents |
|---|---|
| Table S2 | Nominal and instantiated channel dimensions under the YOLO11n n-scale configuration |
| Code Block S1 | Complete GMS-YOLO11n YAML configuration |
| Table S3 | Instantiated GMS-YOLO11n architecture and layer-wise parameter counts for a 640 × 640 input |
| Table S4 | Tensor shapes at the inputs, intermediate operations, and output of MSAMFusion |
| Table S5 | Module-wise parameter counts and GFLOPs of GMS-YOLO11n for a 640 × 640 input |
| Figure S1 | Framework-generated GMS-YOLO11n model summary |

## Table S2. Nominal and instantiated channel dimensions under the YOLO11n n-scale configuration

| Feature | YAML nominal channels | Actual channels |
|---|---|---|
| P2/4 | 256 | 64 |
| P3/8 | 512 | 128 |
| P4/16 | 512 | 128 |
| P5/32 | 1024 | 256 |
| MSAMFusion embedding | 1024 | 256 |
| Attention embedding | 256 | 64 |
| Attention heads | — | 4 |
| Dimension per head | — | 16 |

## Code Block S1. Complete YAML configuration of GMS-YOLO11n

```
nc: 1
scale: n


scales:
  n: [0.50, 0.25, 1024]
  s: [0.50, 0.50, 1024]
  m: [0.50, 1.00, 512]
  l: [1.00, 1.00, 512]
```

```
  x: [1.00, 1.50, 512]

backbone:
  - [-1, 1, Conv, [64, 3, 2]]
  - [-1, 1, SGBC, [128, 3, 2]]
  - [-1, 2, C3k2, [256, False, 0.25]]
  - [-1, 1, Conv, [256, 3, 2]]
  - [-1, 2, C3k2, [512, False, 0.25]]
  - [-1, 1, SGBC, [512, 3, 2]]
  - [-1, 2, C3k2, [512, True]]
  - [-1, 1, Conv, [1024, 3, 2]]
  - [-1, 2, C3k2, [1024, True]]
  - [-1, 1, SPPF, [1024, 5]]
  - [-1, 2, C2PSA, [1024]]

head:
  - [[2, 4, 6, 8], 1, MSAMFusion, [1024, 4, True, True, 256, 0.0]]
  - [[-1, 10], 1, Concat, [1]]
  - [-1, 2, C3k2, [1024, True]]
  - [-1, 1, nn.Upsample, [None, 2, "nearest"]]
  - [[-1, 6], 1, Concat, [1]]
  - [-1, 2, C3k2, [512, False]]
  - [-1, 1, nn.Upsample, [None, 2, "nearest"]]
  - [[-1, 4], 1, Concat, [1]]
  - [-1, 2, C3k2, [256, False]]
  - [-1, 1, Conv, [256, 3, 2]]
  - [[-1, 16], 1, Concat, [1]]
  - [-1, 2, C3k2, [512, False]]
  - [-1, 1, Conv, [512, 3, 2]]
  - [[-1, 13], 1, Concat, [1]]
  - [-1, 2, C3k2, [1024, True]]
  - [[19, 22, 25], 1, Detect, [nc]]
```

**Table S3. Instantiated GMS-YOLO11n architecture and layer-wise parameter counts for a 640 × 640 input.**

| Layer | From | Module | Instantiated arguments | Parameters |
|---|---|---|---|---|
| 0 | -1 | Conv | [3, 16, 3, 2] | 464 |
| 1 | -1 | SGBC | [16, 32, 3, 2] | 1,874 |
| 2 | -1 | C3k2 | [32, 64, 1, False, 0.25] | 6,640 |
| 3 | -1 | Conv | [64, 64, 3, 2] | 36,992 |
| 4 | -1 | C3k2 | [64, 128, 1, False, 0.25] | 26,080 |
| 5 | -1 | SGBC | [128, 128, 3, 2] | 34,626 |
| 6 | -1 | C3k2 | [128, 128, 1, True] | 87,040 |
| 7 | -1 | Conv | [128, 256, 3, 2] | 295,424 |
| 8 | -1 | C3k2 | [256, 256, 1, True] | 346,112 |

| Layer | From | Module | Instantiated arguments | Parameters |
|---|---|---|---|---|
| 9 | -1 | SPPF | [256, 256, 5] | 164,608 |
| 10 | -1 | C2PSA | [256, 256, 1] | 249,728 |
| 11 | [2,4,6,8] | MSAMFusion | [[64,128,128,256], 256, 4, True, True, 64, 0.0] | 216,192 |
| 12 | [-1,10] | Concat | [1] | 0 |
| 13 | -1 | C3k2 | [512, 256, 1, True] | 411,648 |
| 14 | -1 | Upsample | [None, 2, nearest] | 0 |
| 15 | [-1,6] | Concat | [1] | 0 |
| 16 | -1 | C3k2 | [384, 128, 1, False] | 111,296 |
| 17 | -1 | Upsample | [None, 2, nearest] | 0 |
| 18 | [-1,4] | Concat | [1] | 0 |
| 19 | -1 | C3k2 | [256, 64, 1, False] | 32,096 |
| 20 | -1 | Conv | [64, 64, 3, 2] | 36,992 |
| 21 | [-1,16] | Concat | [1] | 0 |
| 22 | -1 | C3k2 | [192, 128, 1, False] | 86,720 |
| 23 | -1 | Conv | [128, 128, 3, 2] | 147,712 |
| 24 | [-1,13] | Concat | [1] | 0 |
| 25 | -1 | C3k2 | [384, 256, 1, True] | 378,880 |
| 26 | [19,22,25] | Detect | [1, [64,128,256]] | 430,867 |

**Table S4. Tensor shapes at the inputs, intermediate operations, and output of MSAMFusion**

| Stage | Tensor | Input shape | Operation | Output shape |
|---|---|---|---|---|
| Backbone layer 2 | P2 | B x 64 x 160 x 160 | 1 x 1 Conv-BN-SiLU | B x 256 x 160 x 160 |
| Backbone layer 4 | P3 | B x 128 x 80 x 80 | 1 x 1 Conv-BN-SiLU | B x 256 x 80 x 80 |
| Backbone layer 6 | P4 | B x 128 x 40 x 40 | 1 x 1 Conv-BN-SiLU | B x 256 x 40 x 40 |
| Backbone layer 8 | P5 | B x 256 x 20 x 20 | 1 x 1 Conv-BN-SiLU | B x 256 x 20 x 20 |
| Aligned P2 | $\tilde{P}_2$ | B x 256 x 160 x 160 | Nearest-neighbor resize | B x 256 x 20 x 20 |
| Aligned P3 | $\tilde{P}_3$ | B x 256 x 80 x 80 | Nearest-neighbor resize | B x 256 x 20 x 20 |
| Aligned P4 | $\tilde{P}_4$ | B x 256 x 40 x 40 | Nearest-neighbor resize | B x 256 x 20 x 20 |
| Aligned P5 | $\tilde{P}_5$ | B x 256 x 20 x 20 | Identity spatial alignment | B x 256 x 20 x 20 |
| Multi-scale branch | $F_{ms}$ | Four tensors, each B x 256 x 20 x 20 | Element-wise mean | B x 256 x 20 x 20 |
| Query projection | Q | B x 256 x 20 x 20 | 1 x 1 Conv-BN, 256 -> 64 | B x 64 x 20 x 20 |
| Shared key projection | $K_j$ | B x 256 x 20 x 20 | 1 x 1 Conv-BN, 256 -> 64 | B x 64 x 20 x 20 |
| Shared value projection | $V_j$ | B x 256 x 20 x 20 | 1 x 1 Conv-BN, 256 -> 64 | B x 64 x 20 x 20 |

| Stage | Tensor | Input shape | Operation | Output shape |
|---|---|---|---|---|
| Multi-head reshape | Q/K/V | B x 64 x 20 x 20 | 4 heads, 400 tokens, 16 dim/head | B x 4 x 400 x 16 |
| Attention outputs | $A_{5j}$ | Four tensors, each B x 64 x 20 x 20 | Element-wise mean | B x 64 x 20 x 20 |
| Output projection | $F_{att}$ | B x 64 x 20 x 20 | 1 x 1 Conv-BN, 64 -> 256 | B x 256 x 20 x 20 |
| Enhanced feature | $F_{enh}$ | $F_{ms}$ $+F_{att}$ | Element-wise addition | B x 256 x 20 x 20 |
| Gated output | $P_5'$ | $\tilde{P}_5$ and $F_{enh}$ | Channel-wise gated interpolation | B x 256 x 20 x 20 |

**Table S5. Module-wise parameter counts and GFLOPs of GMS-YOLO11n for a 640 × 640 input**

| Layer | Module | Output resolution | Parameters | GFLOPs |
|---|---|---|---|---|
| 0 | Conv | 320 × 320 | 464 | 0.095 |
| 1 | SGBC-1 | 160 × 160 | 1,874 | 0.096 |
| 2 | C3k2 | 160 × 160 | 6,640 | 0.340 |
| 3 | Conv | 80 × 80 | 36,992 | 0.473 |
| 4 | C3k2 | 80 × 80 | 26,080 | 0.334 |
| 5 | SGBC-2 | 40 × 40 | 34,626 | 0.111 |
| 1 and 5 | SGBC total | | 36,500 | 0.207 |
| 6 | C3k2 | 40 × 40 | 87,040 | 0.279 |
| 7 | Conv | 20 × 20 | 295,424 | 0.236 |
| 8 | C3k2 | 20 × 20 | 346,112 | 0.277 |
| 9 | SPPF | 20 × 20 | 164,608 | 0.132 |
| 10 | C2PSA | 20 × 20 | 249,728 | 0.200 |
| 11 | MSAMFusion | 20 × 20 | 216,192 | 1.710 |
| 12 | Concat | 20 × 20 | 0 | 0.000 |
| 13 | Post-fusion C3k2 | 20 × 20 | 411,648 | 0.329 |
| 14 | Upsample | 40 × 40 | 0 | 0.000 |
| 15 | Concat | 40 × 40 | 0 | 0.000 |
| 16 | C3k2 | 40 × 40 | 111,296 | 0.356 |
| 17 | Upsample | 80 × 80 | 0 | 0.000 |
| 18 | Concat | 80 × 80 | 0 | 0.000 |
| 19 | C3k2 | 80 × 80 | 32,096 | 0.411 |
| 20 | Conv | 40 × 40 | 36,992 | 0.118 |
| 21 | Concat | 40 × 40 | 0 | 0.000 |
| 22 | C3k2 | 40 × 40 | 86,720 | 0.278 |
| 23 | Conv | 20 × 20 | 147,712 | 0.118 |
| 24 | Concat | 20 × 20 | 0 | 0.000 |
| 25 | C3k2 | 20 × 20 | 378,880 | 0.303 |
| 26 | Detect | P3/P4/P5 | 430,867 | 1.698 |
| — | **Complete GMS-YOLO11n** | — | 3,101,991 | 7.90 |

**Figure S1. Framework-generated layer summary of the instantiated GMS-YOLO11n model**

```
                   from  n    params  module                                       arguments
  0                  -1  1       464  ultralytics.nn.modules.conv.Conv             [3, 16, 3, 2]
  1                  -1  1      1874  ultralytics.nn.Extramodule.SGBC.SGBC         [16, 32, 3, 2]
  2                  -1  1      6640  ultralytics.nn.modules.block.C3k2            [32, 64, 1, False, 0.25]
  3                  -1  1     36992  ultralytics.nn.modules.conv.Conv             [64, 64, 3, 2]
  4                  -1  1     26080  ultralytics.nn.modules.block.C3k2            [64, 128, 1, False, 0.25]
  5                  -1  1     34626  ultralytics.nn.Extramodule.SGBC.SGBC         [128, 128, 3, 2]
  6                  -1  1     87040  ultralytics.nn.modules.block.C3k2            [128, 128, 1, True]
  7                  -1  1    295424  ultralytics.nn.modules.conv.Conv             [128, 256, 3, 2]
  8                  -1  1    346112  ultralytics.nn.modules.block.C3k2            [256, 256, 1, True]
  9                  -1  1    164608  ultralytics.nn.modules.block.SPPF            [256, 256, 5]
 10                  -1  1    249728  ultralytics.nn.modules.block.C2PSA           [256, 256, 1]
MSAMFusion instantiated with actual channels: inputs=[64, 128, 128, 256], embed_dim=256, attn_dim=64, heads=4, head_dim=16.
 11        [2, 4, 6, 8]  1    216192  ultralytics.nn.Extramodule.MSAMFusion.MSAMFusion[[64, 128, 128, 256], 256, 4, True, True, 64, 0.
0]
 12            [-1, 10]  1         0  ultralytics.nn.modules.conv.Concat           [1]
 13                  -1  1    411648  ultralytics.nn.modules.block.C3k2            [512, 256, 1, True]
 14                  -1  1         0  torch.nn.modules.upsampling.Upsample         [None, 2, 'nearest']
 15             [-1, 6]  1         0  ultralytics.nn.modules.conv.Concat           [1]
 16                  -1  1    111296  ultralytics.nn.modules.block.C3k2            [384, 128, 1, False]
 17                  -1  1         0  torch.nn.modules.upsampling.Upsample         [None, 2, 'nearest']
 18             [-1, 4]  1         0  ultralytics.nn.modules.conv.Concat           [1]
 19                  -1  1     32096  ultralytics.nn.modules.block.C3k2            [256, 64, 1, False]
 20                  -1  1     36992  ultralytics.nn.modules.conv.Conv             [64, 64, 3, 2]
 21            [-1, 16]  1         0  ultralytics.nn.modules.conv.Concat           [1]
 22                  -1  1     86720  ultralytics.nn.modules.block.C3k2            [192, 128, 1, False]
 23                  -1  1    147712  ultralytics.nn.modules.conv.Conv             [128, 128, 3, 2]
 24            [-1, 13]  1         0  ultralytics.nn.modules.conv.Concat           [1]
 25                  -1  1    378880  ultralytics.nn.modules.block.C3k2            [384, 256, 1, True]
 26        [19, 22, 25]  1    430867  ultralytics.nn.modules.head.Detect           [1, [64, 128, 256]]
GMS-YOLO11n summary: 270 layers, 3,101,991 parameters, 3,101,975 gradients, 7.9 GFLOPs
```
